# gEDWIN: a simple and practical index for real-time monitoring of emergency department crowding

**DOI:** 10.1186/s12873-025-01397-4

**Published:** 2025-11-25

**Authors:** Hwan-Jin Yoon, Justin Boyle, Ibrahima Diouf, Vahid Riahi, Hamed Hassanzadeh, Sankalp Khanna

**Affiliations:** 1 Australian e-Health Research Centre, Commonwealth Scientific and Industrial Research Organisation, Melbourne, Victoria 3052 Australia; 2 Australian e-Health Research Centre, Commonwealth Scientific and Industrial Research Organisation, Brisbane, Queensland 4029 Australia

**Keywords:** Emergency department crowding, Emergency department work index, Bed ratio, Occupancy ratio, Acuity ratio, Generalised emergency department work index

## Abstract

**Purpose:**

To propose a generalised Emergency Department Work Index (gEDWIN) as an alternative to the traditional Emergency Department Work Index (EDWIN), which is impractical for real-time warning due to its complexity and data requirements. gEDWIN offers a more efficient, adaptable, and scalable approach for monitoring emergency department (ED) crowding in real time.

**Methods:**

The relationships between various emergency department crowding indices—including Bed Ratio (BR) or Occupancy Rate (OR), Acuity Ratio (AR), and EDWIN—are thoroughly investigated. By leveraging these relationships, we propose a new gEDWIN. Currently, BR is considered one of the most effective ED crowding indices. A simulation study was performed to validate the effectiveness of the gEDWIN. A comprehensive comparison between BR (or OR), gEDWIN, and the traditional EDWIN was conducted to evaluate their performance using real Emergency Department data from three hospitals (two large and one medium-sized) in Australia.

**Results:**

The results demonstrated that gEDWIN performed well. Despite the lack of standardised measurement tools, gEDWIN provided a promising metric for ED crowding and shows potential for broader applicability across different ED settings. The comparative evaluation indicated that gEDWIN provides performance comparable to EDWIN, while offering a simpler and more practical approach to estimating ED occupancy rate. In addition, gEDWIN distinguishes itself with its simplicity and practicality, allowing for real-time monitoring of ED crowding. Its design makes it suitable for use across various hospital settings, regardless of their size or physician volume.

**Conclusion:**

gEDWIN’s simplicity and practicality suggest it may be a valuable tool for real-time monitoring of ED crowding across hospitals of varying sizes and physician volumes. However, its broader adoption across diverse healthcare environments will require further validation in varied clinical contexts.

**Clinical trial number:**

Not applicable.

**Supplementary information:**

The online version contains supplementary material available at 10.1186/s12873-025-01397-4.

## Introduction

Emergency department (ED) crowding is a pressing issue globally, arising when the demand for emergency services exceeds available resources. This crisis adversely affects both patients and healthcare providers. Unfortunately, assessing the impact of adaptive strategies and potential solutions is challenging due to the lack of a clear definition and consensus on a universal metric of crowding.

Traditionally, crowding was estimated by the frequency of ambulance diversion or an evaluation of the number of patients leaving without being seen (LWBS). However, such measures were not very reliable due to variations in criteria triggering ambulance diversion among different EDs. The lack of standard measures for ED crowding makes it difficult to determine the causes and consequences of ED crowding across institutions [1, 2]. As a result, numerous quantitative measures have been proposed but there is no universally accepted quantitative index of ED crowding, and ED crowding remains difficult to define. Previous work evaluating crowding measures showed that the complex quantitative measures were performed well but none of them were better than the simple Occupancy Rate (OR).

In the literature, various indicators have been explored to measure ED crowding. Among these, the Emergency Department Work Index (EDWIN) is considered one of the most efficient, incorporating different parameters related to patient management in the ED [3, 4].

This study critically examines the validity of the EDWIN as a measure of ED crowding, considering its efficiency in incorporating various parameters related to patient management. However, recognizing the need for a standardized approach, we propose the generalised Emergency Department Work Index (gEDWIN). This new index aims to provide a more comprehensive and universally applicable metric for ED crowding by refining existing parameters and integrating additional factors and to conduct a comparative evaluation of ED OR and gEDWIN using real ED data from two large and one medium sized hospital in Australia.

## Methods

### Measurement of ED crowding

The bed ratio (BR) or the OR, the acuity ratio (AR), and EDWIN measures were considered [5–7]. First, the relationships between EDWIN, OR and AR were investigated and then by leveraging these relationships, we proposed the gEDWIN.

#### Bed ratio and occupancy rate

The BR quantifies the relationship between the number of ED patients, $B_A$, and the number of treatment spaces in ED, $B_T$, at a given point in time. The BR considers the current number of ED patients, the predicted patient arrivals, $Pred.Arr$, and departures for the next hour, $Pred.Dep$, and the total number of ED treatment spaces. The BR is given by

1 $$ \textrm{BR} = \frac{B_A + \left( Pred.Arr- Pred.Dep\right)}{B_T} .$$

Predicting the number of patient arrivals and departures for the next hour is challenging due to significant variability between hospitals. Factors such as nonlinearity in hourly patterns, fluctuations in arrivals and departures, and emergency patients arriving by ambulance contribute to this difficulty. Therefore, in calculating the BR, it is not readily practicable to include the number of predicted arrivals and departures. Additionally, there is no standard method for predicting patient arrivals and departures. Hence, we assume that the predicted number of arrivals and departures for the next hour in the ED are the same, $\left( Pred.Arr- Pred.Dep\right) = 0$. Consequently, the BR (1) becomes the ED OR, which is simply the ratio of the number of patients in the ED to the total number of ED treatment spaces (beds):

2 $$ \textrm{BR} = \textrm{OR} = \frac{B_A}{B_T}$$

If $\textrm{OR} > 1$, it indicates ED overcrowding.

#### Acuity ratio

The AR uses triage data to calculate the relative burden of illness in the ED at any one particular point in time. The analysis requires a numerical triage category with the highest number assigned to the most acutely ill patients. Let assume triage categories 1 through 5. The AR is the average acuity of all patients in the ED and is given by

3 $$ \textrm{AR} = \sum_{i=1}^{5} \frac{(n_i \times t_i)}{B_A}$$

where $n_i$ is the number of patients present in the ED in triage category $i$, and $t_i$ is the triage scale (Emergency Severity Index, ESI).

The proportion of ED patients in triage category $i$, $n_i / B_A$, represents the weight of that category within the ED patients. Hence, the Eq. (3) can be rewritten as

4 $$ \textrm{AR} = \sum_{i=1}^{5} \frac{(n_i \times t_i)}{B_A} = \sum_{i=1}^{5} (w_i \times t_i),$$

where $w_i = n_i / B_A, ~ 0 \le w_i \le 1$ and $\sum w_i = 1$, the weight of the category $i$ within the ED patients.

The AR is designed to measure the burden of illness currently faced by the ED. An AR of 1 indicates a low burden of illness, while an AR of 5 indicates a severe burden of illness. The range of the AR depends on the level of triage categories. The ratio of $n_i$ to the number of patients in ED is the weight of the burden of illness for triage category $i$. This weight reflects the proportion of patients in each category relative to the total number of patients in the ED, contributing to the overall AR.

#### Emergency department work index

The EDWIN is defined as

5 $$ \textrm{EDWIN} = \sum_{i=1}^{5} \frac{(n_i \cdot t_i)}{N_a \cdot (B_T - B_A)}$$

where $n_i$ and $t_i$ are the number of patients in the ED in the triage category $i$ and the triage scale, respectively. $N_a,~ B_T,~\textrm{and}~ B_A$ are the number of attending physicians on duty, the number of treatment spaces (beds), and the number of patients in the ED, respectively.

The EDWIN can be demarcated into three parts: (i) $\textrm{EDWIN} < 1.5$ for active but manageable ED, (ii) $1.5 \le \textrm{EDWIN} \le 2$ for busy ED, and (iii) $\textrm{EDWIN} > 2$ for crowded ED [5].

**Note.** There are limitations in the EDWIN. When the number of treatment spaces equals the number of patients in the ED ($B_T=B_A$), EDWIN becomes undefined as the denominator of the EDWIN formula becomes zero. Additionally, if the number of patients in the ED exceeds the number of treatment spaces $(B_T < B_A)$, EDWIN values become negative, which contradicts the expectation that EDWIN should always be positive. These issues have not been adequately addressed in existing explanations.

The EDWIN (5) can be rewritten using other indices such as OR (2) and AR (3) – Modified EDWIN. This reformulation as the combination of the odds ratio of the OR and AR with the reciprocal of the number of physicians provides a clearer and more practical approach to measuring ED crowding:

6 $$\begin{aligned} \textrm{EDWIN} &= \sum_{i=1}^{5} \frac{(n_i \cdot t_i)}{N_a \cdot (B_T - B_A)} \nonumber \\ & = \frac{1}{N_a} \sum_{i=1}^{5} \frac{(n_i \cdot t_i)}{B_T (1 - B_A/B_T)} \nonumber \\ & = \frac{1}{N_a} \sum_{i=1}^{5} \frac{B_A (n_i \cdot t_i)/B_A}{B_T (1 - B_A/B_T)}\\&= \frac{1}{N_a}\cdot \left(\frac{B_A}{B_T}\right)\sum_{i=1}^{5}\frac{(n_i \cdot t_i)/B_A}{(1-B_A/B_T)} ~~~,\textrm{using } (2) \textrm{ and } (3) \nonumber\\ &= \frac{1}{N_a}\cdot \left(\frac{\textrm{OR}}{1- \textrm{OR}}\right)\cdot \textrm{AR} \nonumber\end{aligned}$$

Since an $\textrm{OR} > 1$ indicates that the ED is already overcrowded, we should consider $0 \le \textrm{OR} < 1$ as a real-time warning of impending ED crowding rather than an indication that the ED is already overcrowded. This approach allows for proactive management to prevent the situation from escalating to overcrowding.

#### Generalised emergency department work index

Let $C=1/N_a$, $0 < C \le 1$ as the coefficient of personnel weight and $\textrm{AR}$ (4) as the weighted mean triage score. By taking logarithm in (6), Eq. (6) can be written as

7 $$\begin{aligned} \textrm{gEDWIN} &= \log(\textrm{EDWIN}) \nonumber \\ &= \log\left(C \cdot \left(\frac{\textrm{OR}}{1-\textrm{OR}} \right)\cdot \textrm{AR} \right)\\&= \log C + \log \left(\frac{\textrm{OR}}{1-\textrm{OR}}\right) + \log \textrm{AR} \nonumber\\ &\approx \log \left(\frac{\textrm{OR}}{1-\textrm{OR}}\right) + \log \textrm{AR}. \nonumber\end{aligned}$$

Given that as the OR approaches 1, the odds ratio of OR tends toward infinity, significantly exceeding the constant $C$, we can approximate gEDWIN by neglecting $C$. Additionally, since the AR ranges from 1 to 5, the gEDWIN can be approximated and expressed using the logarithms of the odds ratio of OR and AR as in (7). This simplifies the calculation and provides a practical way to evaluate ED crowding. Additionally, Eq. (7) represents a gEDWIN that is not dependent on the number of physicians. This allows for standardized comparisons between hospitals, as it provides a consistent metric for assessing ED crowding.

From a mathematical perspective, when an EDs occupancy approaches 1, the ‘weight of physicians’, $C$, which scales the effect of physician staffing on ED flow, where $0 < C \le 1$, the effectiveness of C becomes negligible. This happens because the odds ratio associated with the occupancy diverges to infinity, making crowding an overwhelmingly dominant factor that overshadows the influence of other variables like physician staffing. Essentially, the mathematical framework illustrates a point of diminishing returns, where adding more doctors doesn’t significantly improve flow when the ED is critically full.

From a clinical perspective, this mathematical divergence has a clear interpretation: simply increasing the number of physicians in an already severely crowded ED won’t effectively alleviate the crowding. When a ED is at or near its full capacity (occupancy ratio close to 1), the issue is not just about having enough hands-on deck. Even with more physicians, the lack of available treatment spaces, delays in essential support services, or blockages in patient flow to other hospitals mean that doctors cannot actually process patients faster. The core issue is a fundamental capacity and flow problem that additional doctors alone cannot resolve.

### Algorithm for gEDWIN

The development of the gEDWIN follows a logical progression based on established ED crowding metrics in 2.1. The algorithm consists of the following key steps:

**Inputs:**

- $B_A \in \mathbb{N}$: Total number of patients in the Emergency Department (ED)
- $B_T \in \mathbb{N}$: Total number of operational ED beds
- $\mathbf{w} = \{w_1, w_2, ..., w_i \}$: The weight of the triage category $i$ within the ED patient
- $t_i \in \mathbb{N}$: Triage scale (ESI level 1–5)

**Step 1: Compute Bed Occupancy Ratio (OR)**

$$\text{OR} = \frac{B_A}{B_T}$$

**Step 2: Compute Acuity Ratio (AR)**

$$\text{AR} = \sum_{i=1}^{5} (w_i \cdot t_i)$$

**Step 3: Compute gEDWIN**

$$\text{gEDWIN} = \text{logit(OR)} + \text{log(AR)}, $$

$$\text{OR} = \begin{cases}\text{OR}, & \text{if } 0 \le \text{OR} < 1 \\0.99, & \text{Otherwise}\end{cases}$$

where $$\text{logit(OR)}= \text{log(OR/(1-OR))}$$.

**Step 4: Output**

- Return scalar value $\text{gEDWIN} \in \mathbb{R}^+$.

### Summary table

Table 1 presents a summary of the variables and their corresponding formulas used in the ED crowding indices.

**Table 1:** Summary table for ed crowding measures and their formulas

Variable	Formula	Description
Bed ratio (BR)	$(B_A+( Pred.Arr- Pred.Dep))/ B_T$	The relationship between the number of ED patients, $B_A$, and the number of treatment spaces in ED, $B_T$
Occupancy rate (OR)	$B_A / B_T$	The ratio of the number of patients in the ED to the total number of ED treatment spaces (beds)
Acuity ratio (AR)	$\textrm{AR} = \sum_{i=1}^{5} \frac{(n_i \times t_i)}{B_A}$, where $n_i$, the number of patients present in the ED in triage category *i*, and $t_i$ is ESI	The relative burden of illness in the ED at any one particular point in time.
ED Work Index (EDWIN)	$$\textrm{EDWIN} = \sum_{i=1}^{5} \frac{(n_i \cdot t_i)}{N_a \cdot (B_T - B_A)}$$, where $$N_a$$ is the number of physicians on duty in the ED	The EDWIN is demarcated into three parts: : (i) EDWIN $$ < 1.5$$ for active but manageable ED, (ii) EDWIN = [1.5, 2] for busy ED, and (iii) EDWIN $$ > 2$$ for crowded ED
Generalised EDWIN (gEDWIN)	$\textrm{gEDWIN} \approx \log \left(\frac{\textrm{OR}}{1-\textrm{OR}}\right) + \log \textrm{AR}$	Generalised EDWIN

## Results

### Simulation

The descriptions of the parameters of EDWIN for simulation are in Table 2. The number of attending physicians in ED varies from 1 to 5 which can represent the volume of the hospital, i.e., the more physician working in the ED are associated with bigger hospitals. The range of OR is $(0,1)$ which doesn’t include the boundary values. The weighted mean triage score which is the same as AR varies between 1 to 5. Simulation and statistical analysis were carried out using R 4.4.1 [8]. R code to recreate the simulation and analysis has been provided within the supplementary documents.

**Table 2:** Parameters for the simulation

Parameter	Description	Range
$N_a$	The number of physicians in ED	$[1, 5]$
OR	Proportion of $B_A / B_T$	$(0, 1)$
$\textrm{AR} = \sum w_i \cdot t_i$	Weighted mean triage score	$[1, 5]$

We generated 20 values of OR from $[0.01, 0.99]$ and 50 values of AR from $[1,5]$ for each case of the number of doctors in ED, $N_a$. EDWIN and gEDWIN were calculated using (6) and (7), respectively. A detailed R code to recreate the simulation and analysis is provided in the supplementary document for reproducibility.

**Fig. 1:**
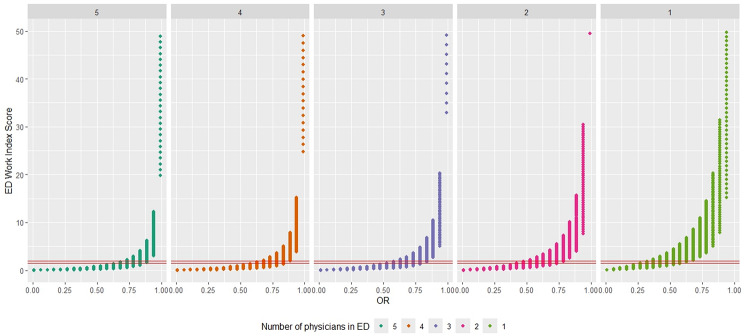
ED work index score by OR: red solid lines - lower (1.5) and upper (2.0) thresholds

Figure 1 illustrates the EDWIN for 5 different ED workload scenarios. The values at the top of the plots indicate the number of doctors in the ED, $N_a$, indicating an inverse relationship with doctor availability. The EDWIN divides into three parts, representing levels of crowding: below the lower red line indicates an “active but manageable” ED, between two red lines signifies a “busy” ED, and above the upper red line denotes a “crowded” ED. As shown in Fig. 1, the measure of EDWIN was very skewed and exponentially increased as OR increased. The EDWIN measure tends to produce values that are too large, making it difficult to see values below 2. The EDWIN scores are heavily influenced by the hospital’s volume and the OR, particularly the odds ratio of OR. As a result, direct comparison between hospitals is challenging unless adjustments are made to the volume of physicians. For $N_a = 1$, when the OR was around 0.4, it indicated a crowded ED. In contrast, with $N_a = 5$, the ED was considered crowded only when the OR was around 0.75.

For comparing ED crowding between hospitals, we used the gEDWIN and established new cut-off values for classifying the levels of crowding. These new cut-off values were aligned with the existing cut-off values for EDWIN (Table 3). The new cut-off were derived from the modified EDWIN, which incorporates occupancy ratio and AR, $(\textrm{OR}/(1-\textrm{OR})) \cdot \textrm{AR} = 1.5 (2) \cdot N_a$, and were further refined using simulation study to closely align with the EDWIN thresholds.

**Table 3:** ED crowding index Cut-off values

ED status	Current Index		Proposed Index
	OR	EDWIN	gEDWIN
Active	-	$$ < 1.5$$	$$ < 2.06$$
Busy	$\le 1$	$1.5 \le$,$\le 2$	$2.06 \le$, $\le 2.34$
Crowded	$$ > 1$$	$$ > 2$$	$$ > 2.34$$

**Fig. 2:**
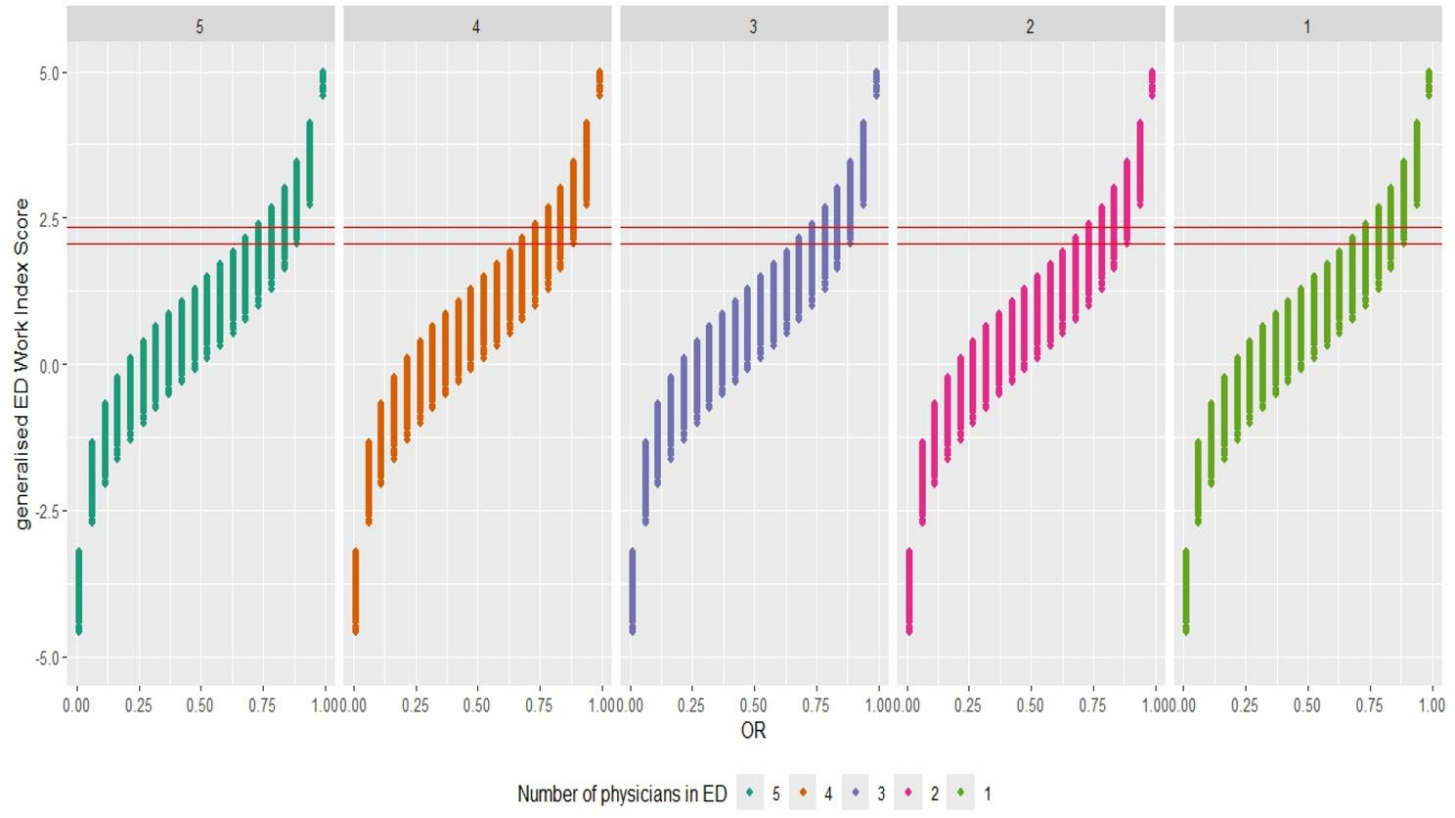
Generalised ED Work Index score by OR: red solid lines - lower (2.06) and upper (2.34) thresholds

As shown in Fig. 2, the gEDWIN enables comparison of ED crowding indices between hospitals regardless of the number of physicians in the ED, demonstrating its robustness. Additionally, the gEDWIN has been appropriately scaled, making it easier to compare ED crowding scores effectively across different hospitals.

### Validation

To evaluate the gEDWIN, ED hourly data collected from three hospitals in Australia, two large and one medium sized, covering the period from 1/1/2018 to 12/31/2018. Hospital A has 83 treatment spaces in the ED made up by Triage area, Resuscitation bays, Acute treatment area (CDU), Minor injuries and fast track, Children’s emergency unit, Short Stay Unit (SSU), and Emergency psychiatry service (EPS). Hospital B has 87 treatment spaces in the ED, while Hospital C, a medium size hospital, has 35 treatment spaces in the ED.

For validation, we used the maximum number of ED spaces denoted as $B_T$, as 83, 87, and 35, corresponding to the capacity of Hospital A’s, Hospital B’s, and Hospital C’s EDs, respectively. The number of patients in ED, $B_A$, and $n_i$ and $t_i$, 1 to 5, were collected hourly and used to calculate the gEDWIN and OR. The OR was used as the gold standard for indicating ED crowding [9]. For computational purposes, when the OR is greater than or equal to 1, it was assigned a maximum value of 0.99. The gEDWIN cut-off values were set in Table 3: (1) Active but manageable ED: $\textrm{gEDWIN} < 2.06$, (2) Busy ED: $2.06 \le \textrm{gEDWIN} \le 2.34$, and (3) Crowded ED: $\textrm{gEDWIN} > 2.34$.

#### Hospital A

**Fig. 3:**
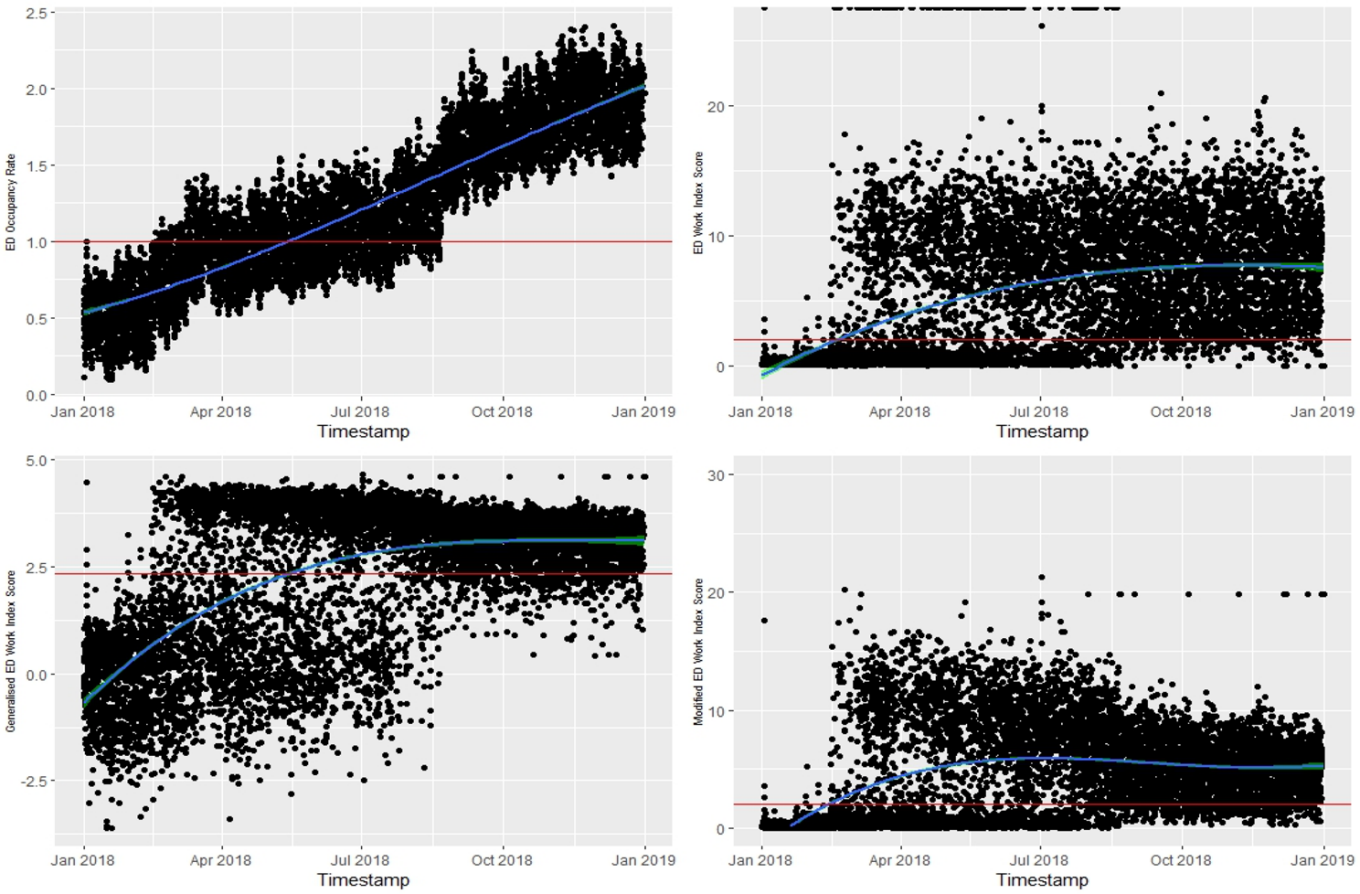
Comparison between ED occupancy rate and ED Work Index score, modified ED Work Index score, and generalised ED Work Index score. Above red line indicates “ED crowding”

In Fig. 3, the top left-hand side of the time-series plot illustrates the trend of the daily ED OR, serving as the gold standard. The plot shows that during the first five months (from January to May), the ED was less crowded. However, from May onwards, the ED remained consistently crowded for the rest of the year. In contrast, the current EDWIN index detected ED crowding slightly earlier than the actual onset. The gEDWIN closely mirrored the trend observed in the ED OR, demonstrating its reliability in reflecting ED crowding.

Table 4 presents the confusion matrix results for three ED crowding indices: EDWIN, Modified EDWIN, and gEDWIN, with accuracy of 0.935, 0.919, and 0.914, respectively, indicating comparable performance. The small differences in precision and recall suggest that all three indices are comparably effective at identifying true positive cases of ED crowding, which is further supported by similar F1 Scores. Detection Rate, the proportion of true positives out of all cases in the dataset, is also comparable across the indices, reinforcing their similar efficacy in detecting ED crowding.

**Table 4:** Confusion matrix table against ED OR

Index	**EDWIN**(5)	**Modified EDWIN**(6)	**gEDWIN**(7)
Accuracy	0.935	0.919	0.914
Precision	0.876	0.825	0.811
Recall	0.885	0.887	0.888
F1 Score	0.881	0.855	0.848
Prevalence	0.270	0.270	0.270
Detection Rate	0.239	0.239	0.240
Detection Prevalence	0.272	0.290	0.295
Balanced Accuracy	0.919	0.909	0.906

**Fig. 4:**
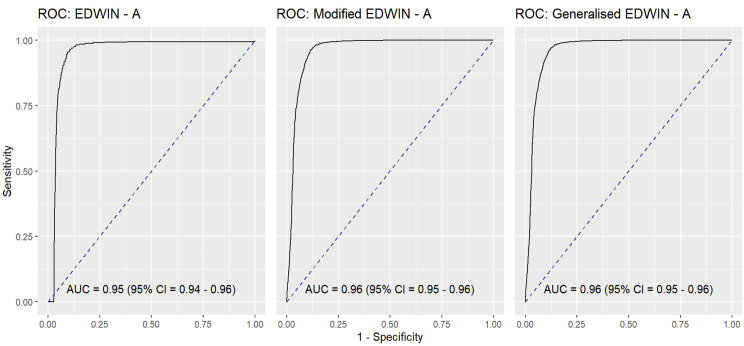
The AUC of the EDWIN, modified EDWIN and generalised EDWIN against OR

To assess the performance of the indices, we conducted the receiver operating characteristic curve (ROC) analysis against OR. The area under the ROC curve (AUC) was used to evaluate index discrimination. Figure 4 depicts the AUC for the EDWIN ($\textrm{AUC} = 0.95$; 95% CI 0.94 to 0.96), modified EDWIN ($\textrm{AUC} = 0.96$; 95% CI 0.95 to 0.96), and gEDWIN ($\textrm{AUC} = 0.96$; 95% CI 0.95 to 0.96).

#### Hospital B

**Fig. 5:**
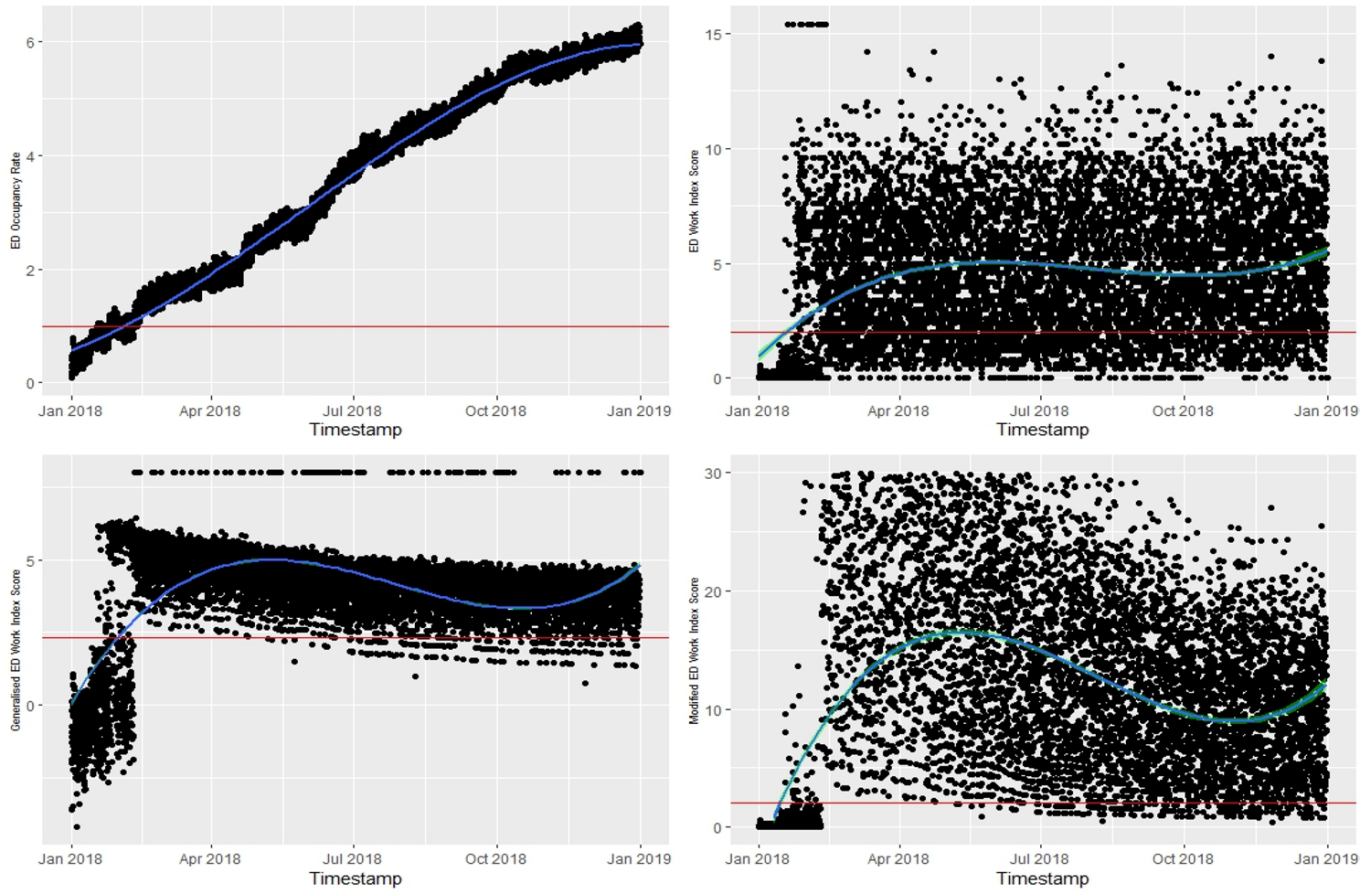
Comparison between ED occupancy rate and ED Work Index score, modified ED Work Index score, and generalised ED Work Index score. Above red line indicates “ED crowding”

In Fig. 5, the top left-hand side of the time-series plot illustrates the trend of the daily ED OR, as the gold standard for ED crowding index. The ED OR plot shows that the first two months (from January to February), the ED was busy not crowded. However, from March onwards, the ED remained consistently crowded for the rest of the year. In this example, although all three indices detected ED crowding a bit earlier than the actual onset, the gEDWIN closely showed the trend observed in the ED OR.

Table 5 presents the confusion matrix results for three ED crowding indices: EDWIN, Modified EDWIN, and gEDWIN, with accuracy of 0.880, 0.982, and 0.980, respectively, indicating promising and comparable performance. The small differences in precision and recall between Modified EDWIN and gEDWIN except for the notably low precision of the EDWIN, indicate that both Modified EDWIN and gEDWIN are similarly effective at identifying true positive cases of ED crowding. This is further supported by their higher F1 Scores compared to the EDWIN.

**Table 5:** Confusion matrix table against ED OR

Index	**EDWIN**(5)	**Modified EDWIN**(6)	**gEDWIN**(7)
Accuracy	0.880	0.982	0.980
Precision	0.422	0.877	0.859
Recall	0.935	0.929	0.932
F1 Score	0.582	0.902	0.894
Prevalence	0.089	0.089	0.089
Detection Rate	0.084	0.083	0.084
Detection Prevalence	0.199	0.095	0.097
Balanced Accuracy	0.905	0.958	0.959

**Fig. 6:**
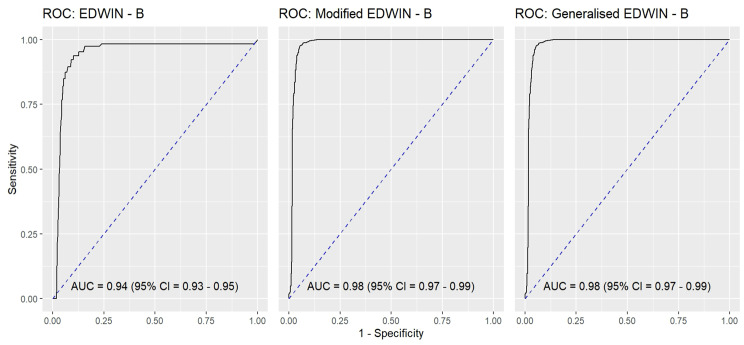
The AUC of the EDWIN, modified EDWIN and generalised EDWIN against OR

Figure 6 shows the AUC for the EDWIN (AUC = 0.94; 95% CI 0.93 to 0.95), Modified EDWIN (AUC = 0.98: 95% CI 0.97 to 0.99) , and gEDWIN (AUC = 0.98: 95% CI 0.97 to 0.99).

#### Hospital C

**Fig. 7:**
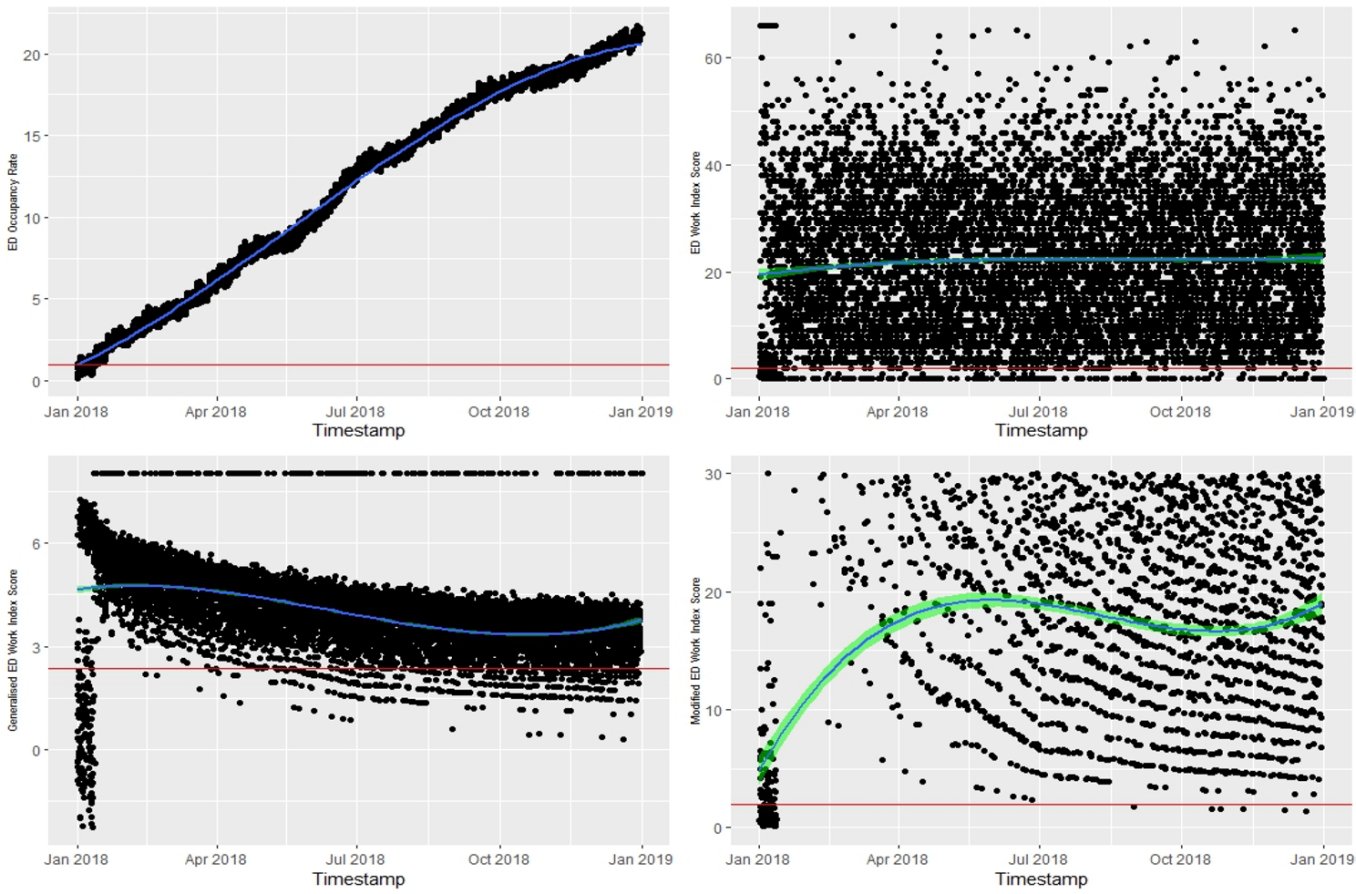
Comparison between ED occupancy rate and ED Work Index score, modified ED Work Index score, and generalised ED Work Index score. Above red line indicates “ED crowding”

In Fig. 7, the top left-hand side of the time-series plot illustrates the trend of the daily ED OR, as the gold standard for ED crowding index. The ED OR plot shows that ED crowding was already evident from January and remained consistently throughout the remainder of the year. In this example, all three indices detected ED crowding in alignment with the ED OR.

Table 6 presents the confusion matrix results for three ED crowding indices: EDWIN, Modified EDWIN, and gEDWIN, with accuracy of 0.966, 0.989, and 0.965, respectively, indicating better performance. The small differences in precision and recall between indices suggest that they are similarly effective at identifying true positive cases of ED crowding. This is further supported by their higher F1 Scores.

**Table 6:** Confusion matrix table against ED OR

Index	**EDWIN**(5)	**Modified EDWIN**(6)	**gEDWIN**(7)
Accuracy	0.966	0.989	0.965
Precision	0.989	0.989	0.996
Recall	0.976	0.999	0.969
F1 Score	0.982	0.994	0.982
Prevalence	0.019	0.019	0.019
Detection Rate	0.008	0.008	0.008
Detection Prevalence	0.031	0.008	0.045
Balanced Accuracy	0.705	0.711	0.873

**Fig. 8:**
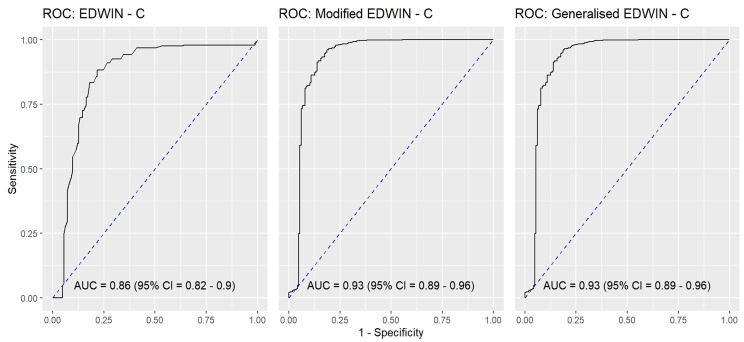
The AUC of the EDWIN, modified EDWIN and generalised EDWIN against OR

Figure 8 shows the AUC for the EDWIN (AUC = 0.86; 95% CI 0.82 to 0.90), Modified EDWIN (AUC = 0.93: 95% CI 0.89 to 0.96) , and gEDWIN (AUC = 0.93: 95% CI 0.89 to 0.96).

### Results

While all three indices perform well, gEDWIN stands out for its simplicity and practicality, making it suitable for real-time monitoring of ED crowding without depending on the number of physicians in ED. This makes gEDWIN a valuable tool for standardising ED crowding measurements and enabling meaningful comparisons across different healthcare facilities. Based on the results from three hospitals, gEDWIN shows strong potential for integration into early warning systems for ED crowding.

## Discussion

This study investigated measures of ED crowding and introduced a new metric called gEDWIN. ED crowding is a critical issue worldwide, occurring when the demand for emergency services exceeds available resources.

The EDWIN was developed to quantify crowding, but it has some limitations. In particular, EDWIN tends to produce skewed results, with values disproportionately increasing as the OR rises. This can make it challenging to interpret lower crowding levels and complicates direct comparisons between hospitals, as the measure is heavy influenced by hospital volume ($B_T - B_A$) and the OR. Therefore, adjusting for physician volume ($N_a$) is often necessary to achieve meaningful comparisons. It is important to note, however, that the EDWIN has undergone extensive validation.

In contrast, gEDWIN addresses these limitations by incorporating a more balanced and normalised approach to measuring ED crowding. As demonstrated in our results, gEDWIN showed promising and comparable performance across institutions. This indicates that gEDWIN may offer more robustness for cross-site comparisons of ED crowding compared to EDWIN.

The advantages of gEDWIN appear promising. With relatively high accuracy rates across the three hospitals, it performs comparably to existing indices in identifying true positive cases of ED crowding. Its relative simplicity and practicality may make it useful for real-time monitoring in healthcare facilities, with potential to support standardised measurements of crowding and inform operational decision-making.

While EDWIN provides valuable insights into ED crowding, gEDWIN enhances the ability to monitor and compare crowding across institutions. Its design suggests improved accuracy and reliability of crowding assessments and indicates potential for use as a real time early warning tool in emergency settings.

To integrate gEDWIN into clinical systems effectively, both data access and system compatibility must be considered. The first step is to establish a reliable data pipeline that ensures the electronic health record (EHR) or ED information system can continuously supply real-time data. This includes the number of patients currently present in the ED and the number of available treatment spaces (beds), which are key variables required for the gEDWIN calculation.

A computational engine must be developed within the clinical IT infrastructure. This can be implemented using R or Python, or through integration with the Hospital’s existing data analytics platform. This module will be responsible for automatically retrieving live ED data, computing the gEDWIN score using the specified formula, and forwarding the result to relevant display or alert systems.

Integration into clinical workflows can occur at multiple points. The gEDWIN score may be incorporated into ED dashboards used by clinicians and administrators to monitor crowding in real-time. It can also be linked to bed management platforms to assist in operational decisions or tied to early warning systems that issue alerts when crowding thresholds are exceeded. Additionally, the score could be presented via mobile applications or internal messaging tools to keep ED leadership informed throughout the day.

In terms of computational efficiency, gEDWIN offers a significant advantage due to its simplicity. The score is derived from basic arithmetic operations that require minimal computational resources, enabling near-instantaneous calculation. This makes the model highly efficient and suitable for implementation in both advanced digital systems and low-resource environments. Therefore, the gEDWIN algorithm can be deployed across multiple clinical sites without straining system performance.

The system can be configured to compute gEDWIN in real time, updating as frequently as every minute, or in batch mode at regular intervals such as hourly, depending on operational needs and IT capacity. Its low computational overhead ensures smooth and scalable integration with a hospital’s broader information systems, making gEDWIN a promising tool for standardising ED crowding assessment and enabling timely responses across diverse healthcare settings.

For example, the dashboard would prominently display the current gEDWIN score, likely colour-coded (e.g., green for low crowding, yellow for moderate, red for severe) and updated every few minutes (30 minutes or hourly), providing an immediate visual cue of the department’s status. Beyond a single score, the dashboard could feature trend lines showing gEDWIN’s progression over the last 12–24 hours, allowing clinicians and administrators to quickly identify developing patterns or sudden surges.

Crucially, this dashboard would be seamlessly linked to bed management platforms. When the gEDWIN score indicates escalating crowding, the system could automatically highlight available inpatient beds or suggest potential areas for patient transfer, thereby assisting in crucial operational decisions to alleviate internal bottlenecks. Furthermore, the gEDWIN score could be integrated into early warning systems, configured to issue automated alerts (e.g., via pop-up notifications on the dashboard, internal messaging tools, or even direct mobile application notifications to ED leadership) when predefined crowding thresholds are exceeded. For instance, a gEDWIN score above a certain critical level might trigger an alert to divert ambulances, activate surge protocols, or request additional staffing.

Beyond the main display, the dashboard could offer drill-down capabilities, presenting the underlying metrics contributing to the gEDWIN score (e.g., waiting room volume, admitted patients boarding in the ED, bed turnaround times). This multi-faceted display, accessible via large monitors in the ED, command centre, or even via dedicated mobile applications, would ensure that all relevant personnel, from frontline clinicians to hospital executives, are continuously informed and empowered to make data-driven decisions to manage ED flow effectively throughout the day.

## Limitations

One of the limitations of the study is that gEDWIN’s effectiveness may depend on the quality and consistency of data across different healthcare facilities, which could affect its applicability in diverse settings. Additionally, while gEDWIN addresses some limitations of EDWIN, it may oversimplify the complexity of ED crowding, potentially overlooking qualitative factors such as patient acuity and service variability. Also, the current model does not incorporate physician-to-patient ratios, which may influence both resource allocation and patient care outcomes. This limitation highlights the need for further investigation to determine how integrating this factor could strengthen the utility of gEDWIN across diverse healthcare settings. Furthermore, we were unable to include the widely used NEDOCS (National Emergency Department Overcrowding Study) index in our analysis due to the unavailability of additional variables. Lastly, in smaller hospitals with fewer ED treatment spaces, crowding may be subject to greater fluctuations, posing challenges for consistent implementation of the ED crowding index.

## Conclusions

The comparative analysis using a confusion matrix and AUC evaluation demonstrated that gEDWIN performed comparably to EDWIN when using ED OR as the reference standard. Despite the absence of standardised measurement tools, gEDWIN appears to be a promising and potentially valuable metric for assessing ED crowding. The evaluation suggests that gEDWIN offers comparable performance to existing measures. Furthermore, gEDWIN’s relative simplicity and practicality make it a potentially suitable option for real-time monitoring of ED crowding across hospitals of different staffing levels. Overall, the preliminary findings indicate that gEDWIN holds promise as a practical and robust tool for ED crowding assessment. However, the limitation of the exclusion of physicians-to-patient ratios underscores the need for further investigation into how this factor could be integrated into gEDWIN to strengthen its applicability. Its broader use also remains contingent on further validation across diverse clinical settings to confirm its reliability and effectiveness under different operational conditions.

## Future work

Our future work will focus on enhancing the robustness, applicability, and depth of ED crowding analysis. Firstly, to address the reliance on data quality and consistency, future research should aim to validate gEDWIN across a more diverse range of healthcare facilities. This would involve a multi-site prospective study to access gEDWIN’s performance in real world, heterogeneous environments. Secondly, to mitigate the potential oversimplification of ED crowding, future study will explore integrating qualitative factors into crowding indices. This could involve developing enhanced models that incorporate specific service demands, and dynamic staff rostering to provide a greater understanding of ED complexity beyond simple occupancy counts. Thirdly, recognising the challenges faced by smaller hospitals, we will investigate tailored solutions for low-volume EDs. This includes developing adaptive crowding indices that account for the greater fluctuations in patient flow and resource availability characteristic of these smaller facilities, ensuring the applicability and utility of crowding metrics across the entire healthcare spectrum. Furthermore, to enrich comparative analysis, a key future direction involves incorporating the NEDOCS index. This would necessitate collaborative efforts with hospitals to access the specific, hospital-dependent variables required for NEDOCS index, hence enabling a direct comparison between gEDWIN, EDWIN, and NEDOCS. Such a comparison would provide valuable insights into the strengths and weakness of each index and inform the development of a more comprehensive ED crowding assessment tool.

## Electronic supplementary material

Below is the link to the electronic supplementary material.

Supplementary Material 1

## Data Availability

Due to privacy regulations, the original data used in this research cannot be publicly shared. However, the simulation data can be reproduced using prescribed parameters, ensuring transparency and replicability of our findings. A detailed R code to recreate the simulation and analysis has been provided in the supplementary document.
