# Supplementary material for: gEDWIN: a simple and practical index for real-time monitoring of emergency department crowding
Source: BMC Emerg Med. 2025 Nov 25;25:244. doi: 10.1186/s12873-025-01397-4 (PMC12648796; doi:10.1186/s12873-025-01397-4)
Supplement: Supplementary file 1 — Supplementary Material 1 [file 12873_2025_1397_MOESM1_ESM.pdf]

Supplementary: gEDWIN: A Simple and Practical Index for Real-Time Monitoring of Emergency Department Crowding

Hwan-Jin Yoon[1*], Justin Boyle[2], Ibrahima Diouf[1], Vahid Riahi[1], Hamed Hassanzadeh[2], Sankalp Khanna[2]

[1*]Australian e-Health Research Centre, Commonwealth Scientific and Industrial Research Organisation, Melbourne, 3052, Victoria, Australia.
[1]Australian e-Health Research Centre, Commonwealth Scientific and Industrial Research Organisation, Melbourne, 3052, Victoria, Australia.
[2]Australian e-Health Research Centre, Commonwealth Scientific and Industrial Research Organisation, Brisbane, 4029, Queensland, Australia.

*Corresponding author(s). E-mail(s): jin.yoon@csiro.au;
Contributing authors: justin.boyle@csiro.au; ibrahima.diouf@csiro.au; vahid.riahi@csiro.au; hamed.hassanzadeh@csiro.au; sankalp.khanna@csiro.au;

1 R codes for simulation

Please note the simulation code is provided on the condition that the original source is acknowledged in any subsequent use or adaptation of the provided code.

```
######################
library(tidyverse)
library(dplyr)
library(forcats)
## Generate Occupancy rate (OR) - 20 cases
pa = seq(0.01,0.99, length = 20)
```

```
## Number of physicians, 1 to 5, Na
## np = 1/Na
np = 1/c(1:5)
np = round(np,3)

## Weighted mean triage score AR = sum (ni*ti)/BA = sum (wi·ti)
## all patients in ED with triage category 1: ni = BA, ti = 5, hence AR = 5 (max)
## all patients in ED with triage category 5: AR = 1 (min) - 50 cases
wmts = seq(1,5,length = 50)
wmts = round(wmts,3)

## Generate Synthetic data
## num_p: number of physicians (num_p = 1:small hospital , num_p = 5: large hospital )
## 1000 for each hospital, total number of samples = 5 *1000 = 5000
edwin_dat = data.frame(num_p = rep(np, each= 1000),
         b_p = rep(rep(pa,each=50),5),
         w_ts = rep(wmts,100))

edwin_dat = edwin_dat %>% mutate(b_odds = b_p/(1-b_p))
edwin_dat$max_w = 5 ## maximum ESI
edwin_dat$min_w = 1 ## minimum ESI

## Calculate EDWIN and gEDWIN
edwin_dat = edwin_dat %>% mutate(EDWIN = num_p*b_odds*w_ts,
                                gEDWIN = log(num_p) + log(b_odds) + log(w_ts))
edwin_dat = edwin_dat %>% mutate(EDWIN_max = num_p*b_odds*max_w,
                                 EDWIN_min = num_p*b_odds*min_w,
                                 gEDWIN_max = log(num_p) + log(b_odds) + log(max_w),
                                 gEDWIN_min = log(num_p) + log(b_odds) + log(min_w))

## Plot x-axis = OR and y-axis = EDWIN
## figure 1 (EDWIN)
edwin_dat$num_p1 = fct_recode(as.factor(edwin_dat$num_p),
                  "1" = "1", "2" = "0.5", "3" = "0.333", "4" = "0.25", "5" = "0.2")

ggplot(edwin_dat, aes(x=b_p, y=EDWIN, group = num_p1, col=num_p1)) +
  geom_point() +
  geom_hline(yintercept = c(1.5,2.0), col='red')+
  ylim(0,100) +
  facet_wrap(~num_p1,nrow=1) + xlab("OR") +
  scale_color_brewer(palette = "Dark2") +
  guides(color = guide_legend(title = "Number of physicians in ED")) +
  theme(legend.position = "bottom")

## figure 2 (gEDWIN)
```

```
ggplot(edwin_dat, aes(x=b_p, y=gEDWIN, group = num_p1, col=num_p1)) +
  geom_point() +
  geom_hline(yintercept = c(c(2.06,2.34)), col='red')+
  ylim(-5,5) + facet_wrap(~num_p1, nrow=1)+ xlab("OR") +
  scale_color_brewer(palette = "Dark2") +
  guides(color = guide_legend(title = "Number of physicians in ED")) +
  theme(legend.position = "bottom")
##################
```

2 Procedure for Real data analysis

- Data Collection - Collect hourly ED presentation data including:
  - ED presentation Date and Time
  - Number of patients for each triage category (e.g., ATS 1-5)
  - Total number of patients currently in the ED
  - Total number of ED spaces (beds available)
  - Number of Physicians in ED - (for EDWIN calculation)
- Calculate OR, AR, EDWIN, and gEDWIN using formulas in the paper (Refer to Section 2.2 in the manuscript) .
